# QUALITY OF COMMUNICATION WITH THE HEALTH CARE TEAM FOR CARE PARTNERS OF PERSONS WITH COGNITIVE IMPAIRMENT

**DOI:** 10.1093/geroni/igz038.749

**Published:** 2019-11-08

**Authors:** Courtney H Van Houtven, Steven Lippmann, Emmanuelle Belanger, Valerie A Smith, Hailey James, Megan Shepherd-Banigan, Brenda Plassman

**Affiliations:** 1 Durham VA HSR&D, Durham, North Carolina, United States; 2 Duke University, Durham, North Carolina, United States; 3 Brown University, Providence, Rhode Island, United States; 4 Duke University/UNC, Durham, North Carolina, United States

## Abstract

The CAregiver Perceptions About CommunIcaTion with Clinical Team members (CAPACITY) instrument measures perceived quality of communication with the health care team and the extent to which caregivers believe that the health care team considers their capacity and preferences in decision-making. A higher score reflects higher perceived quality. This presentation highlights features of CAPACITY scores in a national survey of care partners of persons with unexplained cognitive impairment who sought an Amyloid PET scan. A positive scan (presence of amyloid) indicates probably Alzheimer’s (n=1746). In addition to presenting its psychometric properties in this new sample, discussion focuses on the relationship between CAPACITY scores and a process measure of care that matters – accurate reporting of a new diagnosis. The CAPACITY score reflects an important domain of care quality that could be used more broadly to reflect the caregiver-centeredness of health care experiences and to predict patient outcomes.

